# Targeting Alpha Toxin and ClfA with a Multimechanistic Monoclonal-Antibody-Based Approach for Prophylaxis of Serious *Staphylococcus aureus* Disease

**DOI:** 10.1128/mBio.00528-16

**Published:** 2016-06-28

**Authors:** C. Tkaczyk, M. M. Hamilton, A. Sadowska, Y. Shi, C.S. Chang, P. Chowdhury, R. Buonapane, X. Xiao, P. Warrener, J. Mediavilla, B. Kreiswirth, J. Suzich, C. K. Stover, B. R. Sellman

**Affiliations:** a MedImmune, a member of the AstraZeneca Group, Gaithersburg, Maryland, USA; b Public Health Research Institute, New Jersey Medical School, Rutgers University, Newark, New Jersey, USA

## Abstract

*Staphylococcus aureus* produces numerous virulence factors, each contributing different mechanisms to bacterial pathogenesis in a spectrum of diseases. Alpha toxin (AT), a cytolytic pore-forming toxin, plays a key role in skin and soft tissue infections and pneumonia, and a human anti-AT monoclonal antibody (MAb), MEDI4893*, has been shown to reduce disease severity in dermonecrosis and pneumonia infection models. However, interstrain diversity and the complex pathogenesis of *S. aureus* bloodstream infections suggests that MEDI4893* alone may not provide adequate protection against *S. aureus* sepsis. Clumping factor A (ClfA), a fibrinogen binding protein, is an important virulence factor facilitating *S. aureus* bloodstream infections. Herein, we report on the identification of a high-affinity anti-ClfA MAb, 11H10, that inhibits ClfA binding to fibrinogen, prevents bacterial agglutination in human plasma, and promotes opsonophagocytic bacterial killing (OPK). 11H10 prophylaxis reduced disease severity in a mouse bacteremia model and was dependent on Fc effector function and OPK. Additionally, prophylaxis with 11H10 in combination with MEDI4893* provided enhanced strain coverage in this model and increased survival compared to that obtained with the individual MAbs. The MAb combination also reduced disease severity in murine dermonecrosis and pneumonia models, with activity similar to that of MEDI4893* alone. These results indicate that an MAb combination targeting multiple virulence factors provides benefit over a single MAb neutralizing one virulence mechanism by providing improved efficacy, broader strain coverage, and protection against multiple infection pathologies.

## IMPORTANCE

Alternative strategies to broad-spectrum antibiotics are required to combat the antibiotic resistance epidemic. Previous attempts at active or passive immunization against *Staphylococcus aureus* targeting single antigens have failed in clinical trials despite positive preclinical data. To provide broad disease and isolate coverage, an effective immunization strategy likely must target multiple virulence mechanisms of the pathogen. Herein, we tested a multimechanistic MAb combination targeting alpha toxin (AT) and clumping factor A (ClfA) that neutralizes AT-mediated cytotoxicity, blocks fibrinogen binding by ClfA, prevents bacterial agglutination, targets the bacteria for opsonophagocytic killing, and provides broad isolate coverage in a lethal-bacteremia model. Although each MAb alone was effective in bacteremia against some individual isolates, the MAb combination provided improved protection against other isolates. These results illustrate the importance of targeting multiple virulence mechanisms and highlight the potential for an MAb combination targeting AT and ClfA to effectively prevent *S. aureus* disease.

## INTRODUCTION

*Staphylococcus aureus* is a leading cause of bloodstream infections (BSIs), resulting in ~19,000 deaths annually in the United States (1). An increasing incidence of antibiotic resistance, combined with a greater understanding of the link between disorders resulting from broad-spectrum-antibiotic-mediated disruption of the healthy microbiome, has led to the consideration of pathogen-specific treatment strategies, such as monoclonal antibodies (MAbs) targeting bacterial virulence determinants to prevent or treat serious bacterial infections (2, 3). Because *S. aureus* differentially regulates numerous virulence determinants to cause disease (e.g., adhesins, toxins, immune evasion factors), it is likely that a successful immunotherapeutic strategy for all its potential disease manifestations will be multispecific and may include antibodies that neutralize toxins, block immune evasion mechanisms, prevent bacterial adhesion, and promote opsonophagocytic killing (OPK). The need to target multiple virulence factors is supported by preclinical data demonstrating that vaccination with multiple antigens provides benefit over a single antigen to prevent *S. aureus* bacteremia, but treatment has yet to be tested with monoclonal antibodies (4, 5).

Alpha toxin (AT) is a key virulence factor in several *S. aureus* diseases, including pneumonia, skin and soft tissue infections (SSTI), and bacteremia (6–8). In fact, passive immunization with anti-AT MAbs reduced disease severity in pneumonia and dermonecrosis models (9–11), and vaccination with an AT toxoid with an H35L mutation (AT_H35L_) protected against death in mouse lethal bacteremia and pneumonia models (6, 8, 9, 11–13). AT contributes to multiple aspects of *S. aureus* pathogenesis during bacteremia and sepsis, including stimulating a hyperinflammatory response characteristic of sepsis and activating ADAM10-mediated cleavage of endothelial tight junctions, leading to a loss in vascular integrity (14–16). AT has also been demonstrated to target platelets, which prevents repair of the injured endothelial barrier and promotes organ dysfunction through platelet-neutrophil aggregate formation (17). The role of AT in various aspects of sepsis highlights the potential of an AT-neutralizing MAb to prevent or treat this serious disease.

Among the many *S. aureus* surface adhesins, clumping factor A (ClfA) has been demonstrated to play an important role in serious bloodstream infections (18, 19). ClfA binds fibrinogen and facilitates both bacterial adherence to fibrinogen and bacterial clumping, both of which are key attributes in the development of an *S. aureus* bloodstream infection (20–22). ClfA bound to fibrin or fibrinogen at a site of injury or coated on an indwelling device can facilitate bacterial colonization (18) and bacterial clumping, which is thought to enhance bacterial invasiveness (22–25). ClfA has also been reported to impair complement deposition required for OPK (26). Consistent with these observations, isogenic Δ*clfA* mutants exhibited reduced virulence in infection models (23, 27, 28) and passive immunization with human anti-ClfA-enriched intravenous (i.v.) immunoglobulin (Ig) (Veronate) or an MAb (12-9 or Aurexis) improved disease outcomes for patients with *S. aureus* bloodstream infections (29, 30). However, these antibody preparations failed to improve outcomes in clinical studies of prophylaxis or adjunctive therapy with vancomycin to prevent or treat *S. aureus* bacteremia in very-low-birth-weight infants (31–33). There are also conflicting reports of the value of targeting ClfA alone by active immunization (34, 35).

Given the distinct roles of AT and ClfA in *S. aureus* bloodstream infections, we hypothesized that neutralizing both virulence factors with high-affinity MAbs might provide benefit over prophylaxis with the individual MAbs in *S. aureus* bacteremia. Herein, we report on the identification of an anti-ClfA IgG (11H10) that prevents fibrinogen binding, inhibits bacterial agglutination, promotes OPK, and protects mice from a lethal *S. aureus* bacteremia. Additionally, passive immunization with 11H10 combined with an anti-AT MAb, MEDI4893*, provided enhanced efficacy and broader strain coverage than either MAb alone. These results suggest that immunoprophylaxis with an anti-ClfA–anti-AT MAb combination may provide a prophylactic approach superior to that using the individual MAbs for prevention of serious *S. aureus* bloodstream infections.

## RESULTS

### Alpha-toxin and ClfA are key virulence factors in a mouse lethal bacteremia model.

**FIG 1:**
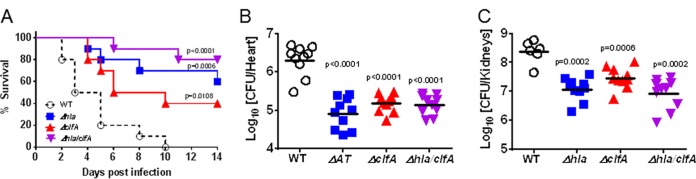
ClfA and AT contribute to virulence in lethal bacteremia in mice. Mice (*n =* 10) were i.v. infected with 6 × 10^7^ CFU of wild-type (WT) SF8300 or an isogenic *ΔclfA*, Δ*hla*, or *ΔclfA Δhla* mutant. (A) Survival was monitored for 2 weeks postinfection. Statistical differences from wild-type SF8300 were analyzed with a log rank (Mantel Cox) test. Bacterial burdens in hearts 14 h postinfection (B) and in kidneys 48 h postinfection (C) were determined. Horizontal lines represent geometric mean numbers of CFU. Statistical differences from the SF8300 wild type were analyzed with a Mann-Whitney *U* test. Data were considered statistically different if *P* was <0.05. Results are representative of three independent experiments.

To confirm a role for AT and ClfA in an *S. aureus* lethal bacteremia model, mice were i.v. infected with the wild-type (WT) community-acquired methicillin-resistant *S. aureus* (CA-MRSA) strain SF8300 or its isogenic Δ*hla*, Δ*clfA*, or Δ*hla* Δ*clfA* mutant. Consistently with published results, infection with the Δ*hla* or Δ*clfA* mutant attenuated disease severity. The double (Δ*clfA* Δ*hla*) mutant had a slight survival benefit over the individual mutants in this model in the same bacterial genetic background (Fig. 1A). Similarly, bacterial numbers were significantly reduced in the hearts of animals (*P* < 0.0001 versus WT SF8300) 14 h postinfection with each of the mutants compared to numbers in the hearts of animals infected with WT SF8300 (Fig. 1B). Numbers of bacterial CFU in the kidneys were also significantly reduced 48 h after infection with each of the mutants relative to numbers of CFU of WT SF8300 (*P* ≤ 0.0006) (Fig. 1C). These results indicate that both AT and ClfA play a role in this model and may be viable targets for immunoprophylaxis against *S. aureus* bacteremia and sepsis.

### Isolation and characterization of the anti-ClfA MAb 11H10.

**FIG 2:**
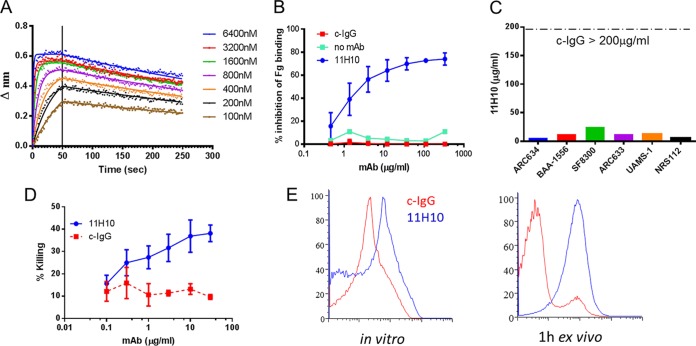
*In vitro* characterization of human anti-ClfA MAb and 11H10. (A) Antibody affinity to ClfA determined with Octet. (B) Inhibition of fibrinogen (Fg) binding. ClfA binding to fibrinogen was measured in the presence of serially diluted 11H10 (300 to 0.03 µg/ml) and c-IgG or in the absence of any MAb. Data are the mean values ± standard deviation (SD) from three independent experiments. (C) Agglutination of *S. aureus* clinical isolates in the presence of human plasma. The graph illustrates the minimal concentration of 11H10 required to inhibit bacterial agglutination. A human negative-control MAb, c-IgG, did not show any inhibitory effect up to 200 µg/ml. Data are representative of three independent experiments, with the same donor as a plasma source. (D) Bacterial OPK assay. *S. aureus* strain Newman was incubated with human HL-60 cells, human sera, and serial dilutions of 11H10 or negative-control IgG. The graph represents mean values ± SD from three independent experiments. (E) 11H10 binding (blue lines) to Δ*spa* SF8300 grown to stationary phase in TSB (left) or *ex vivo* after passage in mice for 1 h (right) was measured by fluorescence-activated cell sorting (FACS). Histograms represent the typical binding of 11H10 compared to that of negative-control c-IgG (red lines).

We previously reported on a potent anti-AT MAb, MEDI4893*, capable of protecting animals against *S. aureus* pneumonia and skin infections (9, 10). This MAb is the precursor to clinical candidate MEDI4893, which is currently in a phase 2b trial for the prevention of *S. aureus* pneumonia in ventilated patients (http://www.clinicaltrialsregister.eu). In the present study, we wanted to compare the protective activity of MEDI4893* to that of a highly potent antibody directed against ClfA in the *S. aureus* sepsis model. To this end, an anti-ClfA MAb was generated using hybridoma technology in VelocImmune mice immunized with the ClfA fibrinogen-binding domain ClfA_N2N3_ (9, 36–38). VelocImmune mice allow for easy conversion to a human IgG1. MAb 11H10 was selected from a panel of 15 anti-ClfA MAbs based on its superior performance in functional assays, which we hypothesized would translate into enhanced protection during infection (e.g., inhibition of fibrinogen binding and bacterial agglutination, binding to *S. aureus* ex vivo, and OPK activity). 11H10 kinetics of binding to ClfA_N2N3_ were measured to determine 11H10 affinity for its target antigen. Association and dissociation constants for ClfA_N2N3_ were determined to be 11.4 × 10^5^ (1/M/s) and 4.8 × 10^−3^ (1/s), respectively, with an estimated *K_D_* (equilibrium dissociation constant) of 4.2 nM (Fig. 2A).

During a bloodstream infection, ClfA binding to fibrinogen facilitates *S. aureus* attachment to fibrinogen-coated surfaces (e.g., an indwelling catheter) and also promotes bacterial agglutination in plasma, a key virulence mechanism reported to result in an invasive phenotype during bloodstream infections (39, 40). 11H10 inhibited ClfA_N2N3_ binding to fibrinogen in a dose-dependent manner (50% inhibitory concentration [IC_50_] = 0.8 µg/ml) (Fig. 2B) and blocked *S. aureus* agglutination in human plasma at a MIC of ≤25 µg/ml (Fig. 2C).

11H10 anti-ClfA OPK and bacterial-clearance activities were evaluated by incubating the bacteria with the MAb in the presence of the human monocytic cell line HL-60 and human serum as a complement source. A collection of *S. aureus* clinical isolates representing diverse ClfA sequences was tested (19). 11H10 exhibited dose-dependent OPK activity against all tested isolates (Fig. 2D; see also Fig. S1 in the supplemental material).

For a therapeutic MAb to be effective, the target antigen must be expressed and the MAb’s epitope must be conserved and accessible to binding *in vivo*. To address this, 11H10 binding was assessed by flow cytometry on 24 different *S. aureus* clinical isolates recovered from the blood of infected mice. Surface binding to ClfA was considered positive if >50% of bacteria exhibited at least a 1-log shift in mean fluorescence compared to that exhibited by bacteria in mice given control human IgG (c-IgG) (Fig. 2E). 11H10 binding was detectable on 19/24 isolates following *in vitro* growth but bound 24/24 clinical isolates recovered from the bloodstream of infected mice (see Table S1 in the supplemental material). These results confirmed that ClfA is differentially regulated among clinical isolates and that the 11H10 epitope is conserved and accessible following *in vivo* passage in mice. Taken together with results from the above-described functional assays, these data indicated that 11H10 was a promising candidate anti-ClfA MAb that binds *S. aureus* passaged *in vivo*, neutralizes fibrinogen binding, and mediates OPK.

### Anti-AT and anti-ClfA protection in CA-MRSA USA300-induced lethal bacteremia.

**FIG 3:**
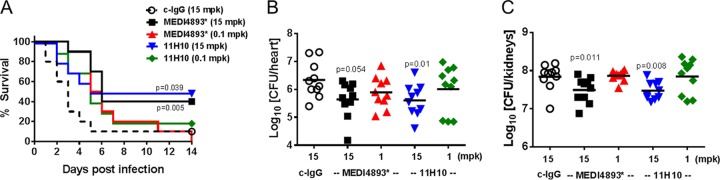
Activities of anti-AT and anti-ClfA in an SF8300 lethal-bacteremia model. (A) BALB/c mice (*n =* 30) were immunized i.p. with MEDI4893*, 11H10, or c-IgG at the indicated concentrations 24 h prior to i.v. challenge with SF8300’s LD_90_ (5e7 CFU), and survival (*n =* 10) was monitored for 2 weeks. mpk, mg/kg. Ten animals were euthanized after 14 h and 48 h for bacterial enumeration in their hearts (B) and kidneys (C). Horizontal bars represent geometric mean numbers of CFU. Statistical differences from the c-IgG group were analyzed with a log rank (Mantel Cox) test, and values were considered statistically different if *P* was <0.05. Data are representative of three independent experiments.

Mice were passively immunized with 11H10 or MEDI4893* 24 h prior to i.v. challenge with a lethal dose of SF8300 and monitored for survival for 14 days to evaluate relative protective activity in a lethal bacteremia model (Fig. 3A). Bacterial burden was measured in the hearts and kidneys. Both 11H10 and MEDI4893* prophylaxis resulted in a dose-dependent increase in survival and significantly reduced numbers of CFU in the hearts and kidneys of infected mice compared to those in c-IgG-administered mice (Fig. 3B and C), indicating that both MAbs were functionally active in this model.

### Anti-ClfA OPK is required for full 11H10 activity *in vivo.*

**FIG 4:**
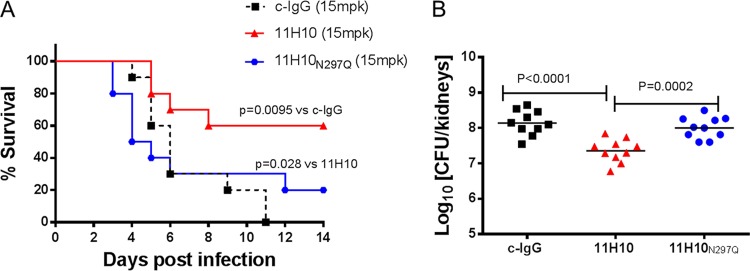
OPK is required for 11H10 efficacy in lethal bacteremia. (A) BALB/c mice (*n =* 10) were injected i.p. with 15 mg/kg (mpk) of 11H10, 11H10_N297Q_, or c-IgG 24 h prior to i.v. challenge with SF8300 (5e7 CFU), and survival was monitored for 2 weeks. Statistical analysis was performed with a log rank test (Mantel Cox test). (B) Bacteria were enumerated in kidneys 48 h after infection. Horizontal lines represent geometric mean numbers of CFU. Statistical analyses were conducted with a Mann-Whitney *U* test. Data are representative of three independent experiments.

We previously showed that MEDI4893* protective activity in a mouse pneumonia model was Fc independent (9). Similarly, Fc function was not required for MEDI4893* protection from lethal bacteremia since MEDI4893*_N297Q_, containing an Fc mutation which abrogates binding to the Fc region of IgG (FcγR) and complement C3a (9, 41, 42), exhibited efficacy similar to what was observed with unmutated MEDI4893* (see Fig. S2 in the supplemental material). An 11H10_N297Q_ Fc mutant was also generated and tested in a lethal-bacteremia model to determine if Fc function is necessary for 11H10 protection in this infection model. As expected, MAb 11H10_N297Q_ exhibited no *in vitro* OPK activity but retained the ability to inhibit bacterial agglutination (Fig. S3). When administered to mice prior to i.v. challenge with different *S. aureus* strains, 11H10_N297Q_ exhibited reduced protective capacity relative to that of 11H10 against 3 different strains (Fig. 4 and S3), indicating that anti-ClfA OPK activity is required for full 11H10-mediated protection in this model.

### An anti-ClfA and anti-AT MAb combination is beneficial in lethal bacteremia.

**FIG 5:**
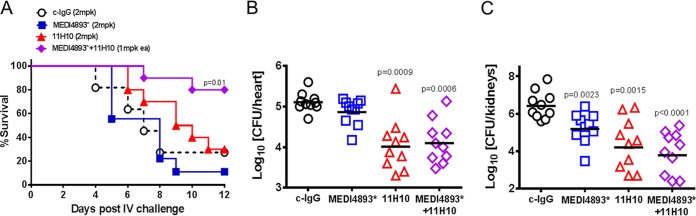
An anti-AT–anti-ClfA MAb combination provides benefit over monotherapy in preventing murine sepsis. Mice (*n =* 10) were immunized i.p. with MEDI4893* (2 mg/kg), 11H10 (2 mg/kg), a combination of both MAbs (1 mg/kg each), or c-IgG (2 mg/kg) 24 h prior to infection, and survival was monitored for 2 weeks. (B and C) Statistical differences from the c-IgG group were analyzed with a log rank (Mantel Cox) test. Bacteria in the heart 14 h postinfection (B) or the kidneys 48 h postinfection (C) were enumerated. Horizontal lines represent geometric mean numbers of CFU. Statistical analysis versus c-IgG was performed with a Mann-Whitney *U* test. Statistical analyses were considered statistically different if *P* was <0.05. Data are representative of three independent experiments.

To determine whether there was benefit from combining anti-AT and anti-ClfA MAb activities, mice were passively immunized with a suboptimal MEDI4893* or 11H10 dose (2 mg/kg of body weight) or a combination of both MAbs (1 mg/kg each) 24 h prior to i.v. challenge with SF8300. Animals were monitored for survival, and bacterial burden was measured in hearts and kidneys. Although prophylaxis with the individual MAbs reduced bacterial burden in one (MEDI4893*) or both (11H10) organs, neither provided a survival benefit relative to that of c-IgG (Fig. 5). In contrast, the MAb combination resulted in significantly increased survival compared to that with c-IgG. In fact, the protective activity seen with a low dose of MAbs in combination exceeded the protection observed with either of the individual MAbs, even when they were administered at a much high dose (compare Fig. 3A and 5A). Although the MAb combination provided a greater survival benefit, the reduction in organ burden observed with the combination was no greater than with 11H10 alone. These results support a previous finding that survival following i.v. challenge with *S. aureus* does not always correlate with bacterial burden at a snapshot in time (17). Overall, our data suggest that prophylaxis with a combination of MAbs having distinct mechanisms of action can provide benefit over individual MAbs in preventing *S. aureus* bloodstream infections.

### An anti-AT–anti-ClfA MAb combination reduces *S. aureus* sepsis-associated markers of organ damage.

**FIG 6:**
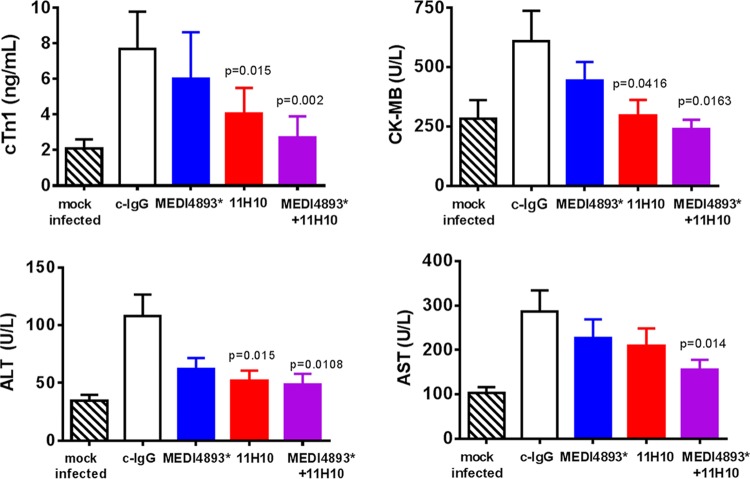
Effect of monotherapy and MAb combination on plasma biomarkers of organ damage. Mice (*n =* 10) were immunized i.p. with MEDI4893* (2 mg/kg), 11H10 (2 mg/kg), a combination of both MAbs (1 mg/kg each), or c-IgG (2 mg/kg) 24 h prior to infection with WT SF8300 (5e7 CFU). Plasma biomarkers of organ damage were measured 48 h after infection. Graphs represent mean values ± standard errors of the means for the biomarkers cardiac troponin 1 in nanograms per milliliter (top left) and for creatine kinase myocardial band (CK-MB) (top right), alanine aminotransferase (bottom left), and aspartate aminotransferase (bottom right) in units per liter. Background levels for each marker were measured in the plasma of mock-infected animals (200 µl PBS i.v.). Statistical analysis relative to c-IgG was performed with Student’s unpaired *t* test. Values were considered statistically different if *P* was <0.05 (*P* values are indicated on the graphs). Data are representative of three independent experiments.

Organ damage is a hallmark of bacterial sepsis and can be monitored by measuring serum levels of cardiac troponin (cTn1), creatine kinase myocardial band (CK-MB), alanine amino transferase (ALT), and aspartate aminotransferase to assess heart and liver damage (43). Animals that received c-IgG prior to *S. aureus* challenge exhibited increased levels of all biomarkers relative to those of mock-infected animals, consistent with sepsis-mediated organ damage (Fig. 6). The biomarkers of organ damage trended lower in anti-AT MEDI4893*-treated animals, but the reductions were not significant. Anti-ClfA 11H10 prophylaxis reduced 3 of 4 (cTn1, CK-MB, ALT) markers, whereas the MAb combination significantly reduced all 4 biomarkers of organ damage compared to c-IgG. These results suggest that while anti-ClfA MAbs can reduce organ damage, the MAb combination provides the most complete protection against sepsis-associated organ damage.

### Anti-AT and anti-ClfA MAb combination provides improved strain coverage.

The efficacy of 11H10 and MEDI4893* against a collection of clinical *S. aureus* isolates representing diverse clonal complexes was tested to evaluate strain coverage. Prophylaxis with 11H10 or MEDI4893* (15 mg/kg) alone significantly increased survival over that with c-IgG following challenge with 6/9 and 3/9 clinical isolates, respectively (Table 1; see also Fig. S4 in the supplemental material). No protection with either MAb alone was observed with two of the isolates (3049057 and NRS261). However, prophylaxis with the MAb combination (7.5 mg/kg each) resulted in a significant increase in survival relative to that with c-IgG following challenge with every isolate tested. These results indicate that although 11H10 and MEDI4893* can each provide protection in an *S. aureus* sepsis model, the protection is strain dependent and the MAb combination provides the greatest isolate coverage.

**TABLE 1:** The anti-AT–anti-ClfA MAb combination provides broad strain coverage in i.v. lethal sepsis^a^

Clinical isolate	*P* value result with:		
	AT MAb (15 mg/kg)	ClfA MAb (15 mg/kg)	MAb combination (7.5 mg/kg each)
2784 (CC1)	+	+	+
NRS382 (CC5)	−	+	+
3049043 (CC5)	−	+	+
4211 (CC5)	+	+	+
SF8300 (CC8)	−	+	+
3049057 (CC3)	−	−	+
NRS261 (CC30)	−	−	+
3049157 (CC30)	+	−	+
3049048 (CC45)	−	+	+

a BALB/c mice (*n =* 10) were injected i.p. with MEDI4893* (15 mg/kg), 11H10 (15 mg/kg), MEDI4893* plus 11H10 (7.5 mg/kg each), or c-IgG (15 mg/kg). Twenty-four hours later, animals were infected i.v. in the tail vein with an LD_90_ of different *S. aureus* clinical isolates from diverse clonal complexes (CC). Survival was monitored for 2 weeks. Results were analyzed with a log rank (Mantel Cox) test. + indicates a *P* value of <0.05, and − indicates a *P* value of >0.05. Each strain was tested at least three times.

### The MEDI4893* and 11H10 combination provides protection in dermonecrosis and pneumonia.

**FIG 7:**
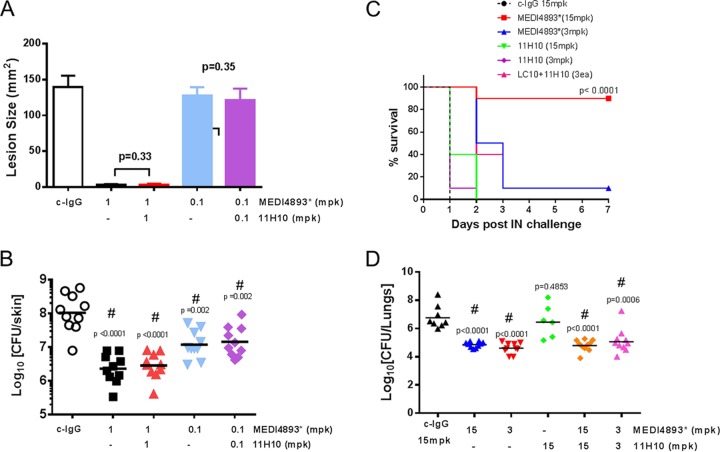
Protection from dermonecrosis and pneumonia with MEDI4893* and the MEDI4893*-11H10 combination. BALB/c mice (*n =* 10) were immunized i.p. with MEDI4893*, a combination of both MEDI4893* and 11H10 MAbs, or c-IgG at the indicated concentrations 24 h prior to intradermal infection with SF8300 (5e7 CFU). (A) The graph represents lesion sizes at day 1 postinfection as mean values ± standard errors. (B) Bacteria in the skin lesions were enumerated 7 days postinfection. Statistical analysis for each group versus c-IgG was performed with a Student unpaired *t* test. (C and D) C57/Bl6 mice (*n =* 10) were injected i.p. with MEDI4893*, 11H10, a combination of both MAbs, or c-IgG at the indicated concentrations 24 h prior to intranasal (IN) infection with SF8300 (1.8e8 CFU). (C) Survival was monitored for 5 days. (D) Bacteria were enumerated in the lungs 24 h postinfection with SF8300 (1e8 CFU). Statistical analysis for each group versus c-IgG was performed with a Student unpaired *t* test. Values were considered statistically different if *P* was <0.05, and statistically significant differences are indicated with a number sign. Data are representative of two independent experiments.

MEDI4893* was previously reported to protect against pneumonia and dermonecrosis in murine infection models (9, 10). We next determined if 11H10 would improve MEDI4893* monotherapy in these two disease models where AT is the major virulence determinant. Passive immunization with MEDI4893* plus 11H10 resulted in lesion sizes similar to those observed in animals passively immunized with MEDI4893* in a mouse dermonecrosis model (Fig. 7A). Similarly, 11H10 combined with MEDI4893* did not improve survival relative to MEDI4893* monotherapy in a pneumonia model (Fig. 7C). Addition of 11H10 also did not potentiate bacterial clearance seen in these models with MEDI4893*, indicating either that ClfA is not expressed or that 11H10 does not effectively promote OPK in this infection context (Fig. 7B and D). These data support previous findings showing that AT is a key virulence determinant in skin and lung infections and indicate that the addition of 11H10 is neither beneficial nor detrimental to the protection observed with MEDI4893* in these models.

## DISCUSSION

Despite positive preclinical data, previous active or passive immunization attempts targeting single *S. aureus* virulence factors (i.e., ClfA, IsdB, or CP5/CP8) failed to prevent *S. aureus* disease in clinical trials (44–46). *S. aureus* expresses a diverse array of virulence factors which contribute to disease pathogenesis (47, 48). Many of these antigens are not highly conserved and can be differentially regulated depending on the site of infection (49–52). Current efforts are shifting to a multiantigen immunization approach to neutralize multiple virulence factors and to provide broad strain coverage (4, 5, 53–55). AT has been reported to be essential for *S. aureus* skin and soft tissue infections (SSTI) and pneumonia (6, 7, 14), and immunization strategies targeting AT reduced disease severity in both models (6, 9–11, 56, 57). Because of its major role in these diseases, AT is currently under development as a stand-alone MAb target for the prevention or treatment of *S. aureus* pneumonia (http://www.clinicaltrialsregister.eu).

AT plays an important role in *S. aureus* bacteremia and sepsis, in addition to SSTI and pneumonia. It contributes to many of the pathogenic effects seen during *S. aureus* bacteremia by disrupting endothelial barriers, altering the coagulation cascade, modifying immune cell function, and contributing to the hyper-inflammatory response in sepsis (17, 58–60). ClfA, another virulence determinant shown to play a role in *S. aureus* bloodstream infections, binds fibrinogen and promotes bacterial agglutination and complement evasion. Active immunization strategies with ClfA, despite generating functional polyclonal opsonic antibodies that also inhibit bacterial binding to fibrinogen *in vitro*, resulted in various levels of protection against multiple strains in bloodstream infection models that may be dependent on the adjuvant and mouse strain (34, 35). Such variability in active immunization strategies highlights the potential benefit of MAb therapy in providing a reliable and potent functional antibody response.

The results presented herein indicate that a combination of anti-AT and anti-ClfA MAbs provides both improved coverage against a representative strain panel and enhanced protective activity. Passive immunization with either the anti-AT or the anti-ClfA MAb protected against challenge with 3 or 6 of 9 *S. aureus* clinical isolates, respectively, and neither MAb provided protection against 2 of the 9 isolates tested. However, the MAb combination provided protection against all strains tested, even with equivalent total MAb doses. Furthermore, passive immunization with subprotective doses of 11H10 (1 mg/kg) plus MED4893* (1 mg/kg) provided strong protection following challenge with CA-MRSA SF8300 (Fig. 5), a strain for which single MAb prophylaxis at a much higher dose (15 mg/kg) was required to provide increased survival in this model (Fig. 3). Taken together, these results suggest that a lack of protection against some isolates by the individual MAbs is not due to a complete lack of antigen expression because the anti-ClfA–anti-AT combination increases survival against multiple strains with apparent synergy against some strains against which neither MAb alone protects even when tested at higher doses (Table 1). These data indicate that neutralizing both AT and ClfA virulence activities with functional MAbs can provide benefit or even synergy relative to that provided by the individual MAbs.

Another multimechanistic MAb approach was shown to provide benefit against *Pseudomonas aeruginosa* in preclinical disease models with a bispecific antibody targeting the cell surface polysaccharide Psl (OPK and anti-cell attachment) and PcrV (anticytotoxic activity) of the type 3 secretion system (61). Combination vaccine approaches to prophylaxis against *S. aureus* disease have been reported; however, the exact mechanisms of action of antibodies generated against each antigen are not clear (4, 5, 62). In our MAb combination, 11H10 blocks ClfA binding to fibrinogen, inhibits bacterial agglutination, and targets the bacteria for OPK (Fig. 2), while MEDI4893* neutralizes the toxic effects of AT (17, 59).

Bacterial antigens targeted by passive or active immunization must be conserved and expressed during infection on circulating clinical isolates. Both of the genes encoding AT (*hla*) and ClfA (*clfA*) are reported to be highly conserved among clinical isolates and are likely expressed in humans, since most individuals tested have IgG titers against these antigens (19, 63–65). These observations are supported by data from mice indicating that both *clfA* and *hla* expression levels increase during a bloodstream infection (49) and ClfA is present on bacteria harvested from the bloodstream of infected mice (Fig. 2E; see Table S1 in the supplemental material) (50). Although humans mount a response against these antigens during natural exposure, the antibodies are generally not thought to be of sufficient quality, activity, and/or quantity to protect from disease (63, 65). Therefore, providing a patient with sufficient quantities of potent, highly functional MAbs may provide benefit over a natural mixed-polyclonal response.

Previous failed attempts at prophylaxis against *S. aureus* have all targeted a single antigen, and none were aimed at neutralizing a secreted toxin (31, 46, 66, 67). Herein, we present data indicating that prophylaxis against *S. aureus* bacteremia with a multimechanistic MAb combination targeting ClfA and AT provides benefit over the individual MAbs by neutralizing multiple virulence mechanisms and targeting the bacteria for OPK. Such an MAb combination may not only extend isolate coverage against *S. aureus* bacteremia but also extend disease coverage beyond SSTI and pneumonia, where AT plays a dominant role. Future clinical studies will be required to further test this hypothesis in human disease.

## MATERIALS AND METHODS

### Bacterial strains.

CA-MRSA SF8300 (USA300) and its isogenic knockout strains for AT (*Δhla*) and ClfA (*ΔclfA*) were previously described (49). The Δ*hla* Δ*clfA* double knockout was constructed in the *Δhla* background as described using the pKOR1 allelic-replacement mutagenesis system and the primers in Table S2 in the supplemental material (49). Reynolds and Wright strains were obtained from the ATCC (Manassas, VA). NRS382 (CC5, MRSA) and NRS261 (CC30, methicillin-susceptible *S. aureus* [MSSA]) were obtained from the Network on Antimicrobial Resistance in *Staphylococcus aureus* (NARSA). 3049043 (CC5, MSSA), 3049057 (CC8, MRSA), 3049157 (CC30, MSSA), and 3049048 (CC45, MSSA) were obtained from Eurofins. Strains ARC4211 (CC5) and ARC2784 (CC1) were obtained from AstraZeneca’s Research Collection. Clonal complex (CC) identifications were determined by protein A typing as previously described (68). Bacteria were grown to mid-log phase (optical density at 600 nm [OD_600_] of 0.8) in tryptic soy broth (TSB; VWR International), washed twice in ice-cold phosphate-bufferd saline (PBS) (Invitrogen), and frozen in 10% glycerol-TSB. Challenge inocula were prepared from one frozen vial diluted in ice-cold PBS to 3 × 10^8^/ml and placed on ice until injection.

### ClfA MAb generation.

Anti-ClfA MAbs were generated by following the repetitive immunization at multiple sites (RIMMS) protocol (69), with slight modifications. Eight-week-old VelocImmune mice were immunized with ClfA containing residues 221 to 559 (ClfA_221–559_) and TiterMax gold adjuvant (Sigma) every 2 to 3 days for 13 days. Lymph nodes were collected and B-cell hybridomas generated after fusion with a P3X myeloma partner. Hybridoma supernatants were first screened for anti-ClfA reactivity by ClfA enzyme-linked immunosorbent assay (ELISA) and *S. aureus* whole-cell ELISA (not shown). Supernatants from the best binders were purified and tested for *in vitro* opsonophagocytic killing (OPK) activity. The most-active hybridomas were then cloned by limiting dilution and tested for fibrinogen binding inhibition, OPK, and *ex vivo* binding to strain SF8300. 11H10 was selected and converted to fully human antibody by grafting a human Fc to the human variable region (37).

### Agglutination inhibition in human plasma.

Six different *S. aureus* clinical isolates were cultured overnight in TSB, washed in PBS, and suspended to 1/10 of the original volume in ice-cold PBS. Anti-ClfA MAb was 2-fold serially diluted starting at 200 µg/ml and mixed with 20 μl of citrated human plasma in a 96-well U-bottom plate (Thermo, Fisher Scientific). Bacteria were added (20 µl) and incubated for 5 min at 37°C. Each well was evaluated visually, and the lowest MAb concentration at which bacteria agglutinated was recorded. R347, a human anti-gp120 MAb (10), was utilized as an isotype control human IgG1 (c-IgG).

### ClfA/fibrinogen binding inhibition assay.

Nunc MaxiSorp plates (Thermo, Fisher Scientific) were coated with 1 µg/ml human fibrinogen (Sigma) overnight at 4°C, washed 3 times with PBS containing 0.1% Tween 20 (wash buffer), and blocked for 1 h at room temperature (RT) with 200 µl/well PBS–1% bovine serum albumin (BSA). Following 3 washes, the plates were incubated for 1 h at RT with a mix of 50 µl ClfA_221–559_ (2 µg/ml) and serial dilutions of anti-ClfA MAb in a 100-µl final volume of PBS. After the washes, bound ClfA was detected using purified rabbit anti-ClfA IgG (5 µg/ml) for 1 h at RT, followed by detection with horseradish peroxidase (HRP)-conjugated goat anti-rabbit IgG Fcγ (1:10,000; Jackson ImmunoResearch Lab) and the 3,3′,5,5′-tetramethylbenzidine (TMB) substrate (KPL). The reaction was stopped after 10 min with 100 µl 0.2 M H_2_SO_4_. Plates were read on a spectrophotometer at an OD_450_. The percentage inhibition of ClfA binding to fibrinogen was calculated with the following formula: 100 − (100 × [OD_ClfA + MAb_]/[OD_ClfA, no MAb_]).

### *Ex vivo* binding assay by flow cytometry.

Six- to eight-week-old female CD1 mice (Harlan) were injected intraperitoneal (i.p.) with 5e8 CFU of *S. aureus*. After 1 or 4 h, cardiac blood was pooled from 4 mice and mixed with ice-cold sodium citrate at a 0.35% final concentration. Eukaryotic cells were lysed with 1% NP-40 (Thermo, Fisher Scientific), and bacteria were recovered after a 5-min centrifugation at 7,000 rpm. The pellet was sonicated in 2 ml ice-cold PBS and washed once in PBS. Bacteria were transferred to a 96-well U-bottom plate (Thermo, Fisher Scientific) and incubated for 30 min at 4°C with rabbit anti-protein A immune sera (1:1,000). The bacteria were then incubated with anti-ClfA MAb or c-IgG (30 µg/ml) for 1 h at 4°C, washed in PBS, and incubated with Alexa 633-conjugated goat anti-human IgG for 30 min at 4°C (Jackson ImmunoResearch Laboratories). Following one wash, live bacteria were stained for 15 min at RT with BODIPY FL vancomycin, and MAb binding was quantified by cytofluorimetry with an LSR II cell analyzer (BD). As controls, *in vitro*-grown bacteria were stained by following the same protocol with anti-ClfA MAb or c-IgG.

### Octet affinity measurement.

11H10 binding kinetics were analyzed using the FortéBio Octet 384 instrument with 384 slanted-well plates (FortéBio). An anti-human Fc biosensor plate (hydrated in kinetic buffer) was first loaded with 1 µg/ml of 11H10 (300 s). The association of purified ClfA in concentrations ranging from 100 to 6,400 nM was measured for 50 s, followed by dissociation into kinetic buffer (FortéBio) (200 s). All steps were performed using a 3-mm sensor offset with 0.6-Hz sensitivity. Data were exported to Prism (GraphPad) for global association/dissociation affinity curve fitting.

### OPK assay

HL-60 cells (ATCC) were differentiated as described previously (70). Cells were washed in saline and adjusted to 1e7 cells/ml in high-glucose Hanks balance salt solution (HG-HBSS) (Invitrogen)–0.1% gelatin (Sigma). Human serum collected from a healthy volunteer was adsorbed against *S. aureus* Reynolds capsule type 5 and *S. aureus* Wright capsule type 8 to deplete endogenous *S. aureus*-specific IgG and used as a complement source (1:100). Clinical isolates were grown overnight in TSB, washed in cold saline, and diluted to 1e6 CFU/ml in saline. Ten microliters of bacteria was incubated on ice for 30 min with 10 µl of serial MAb dilution in 60 µl of HG-HBSS 0.1% gelatin. Ten microliters of sera and 10 µl of HL-60 were then added to the opsonized bacteria. Ten-microliter samples of each well were serially diluted in water–0.1% saponin and dropped on a TSA plate (VWR International) before and after incubation for 1 h at 37°C with 100-rpm orbital shaking. Bacterial colonies were counted after a 16-h incubation of TSA plates at 37°C. The percentage of OPK was calculated as follows: 100 × (100 − [CFU_at 1 h_]/[CFU_at time zero_]).

### Mouse survival and organ burden in sepsis.

Groups of 10 6- to 8-week-old female BALB/c mice (Harlan) were passively immunized by i.p. injection of c-IgG, 11H10, MEDI4893*, or 11H10 plus MEDI4893* and then challenged 24 h later by intravenous (i.v.) injection of the 90% lethal dose (LD_90_) of each *S. aureus* isolate. Survival was monitored over 2 weeks. Statistical analysis of MEDI4893* or 11H10 versus c-IgG was performed with a log rank (Mantel Cox) test. For bacterial enumeration in the hearts and kidneys, animals were euthanized with CO_2_ 14 or 48 h postinfection, respectively. The organs were homogenized in lysis matrix A tubes (VWR International), diluted, and plated for CFU enumeration. Statistical differences between two MAb-treated groups were analyzed with a Mann-Whitney *U* test. Data were considered statistically different if *P* was <0.05, and this is indicated with an asterisk in the figures.

All experiments were performed in accordance with institutional guidelines following experimental protocol review and approval by the Institutional Biosafety Committee (IBC) and the Institutional Animal Care and Use Committee (IACUC) at MedImmune.

### Circulating markers of organ damage.

Cardiac troponin 1 levels were determined by ELISA using a high-density mouse cardiac troponin 1 kit (Life Diagnostics, Inc.). Albumin, alanine aminotransferase (ALT), alkaline phosphatase (ALP), aspartate aminotransferase (AST), and creatine kinase myocardial band (CK-MB) were determined using an AU400 automated clinical chemist analyzer equipped with an ion-selective electrode (Beckman Coulter, Indianapolis, IN). Associated analysis software was operated through a Microsoft Windows NT operating system. Internal quality control materials were analyzed to ensure the precision of the equipment.

### Mouse dermonecrosis and pneumonia models.

Female BALB/c mice (Harlan) were passively immunized i.p. with MEDI4893*, 11H10, or an MAb combination. Dermonecrosis was induced 24 h later with intradermal challenge of SF8300 (5e7 CFU). Lesion sizes and numbers of CFU were measured as previously described (10). Female C57/B6 mice (Jackson) were injected i.p. with a single MAb or a combination of both, and pneumonia was induced by intranasal infection with SF8300 (1e8 CFU) as described previously (9).

## SUPPLEMENTAL MATERIAL

Figure S1  11H10 mediates OPK against multiple clinical isolates. Six different *S. aureus* clinical isolates were grown overnight in TSB medium. Bacteria were opsonized with serial dilutions of 11H10 or c-IgG as a negative control. Differentiated human monocytic cells (HL-60) were added at a 10:1 (HL60-to-bacterium) ratio with human sera at a dilution of 1:100 and with OPK activity measured. The graphs represent OPK killing as means of results from three separate experiments ± standard deviations. Download Figure S1, PDF file, 0.1 MB

Figure S2  Fc function is not required for MEDI4893* protection in lethal bacteremia. BALB/c mice (*n =* 10) were passively immunized i.p. with MEDI4893*, MEDI4893*_N297Q_, or c-IgG at 15 mg/kg and infected 24 h later with SF8300 (5e7 CFU). Survival was monitored for 14 days. Statistical differences between the survival of MEDI4893*- and MEDI4893*_N297Q_-immunized animals and that of c-IgG-immunized animals was analyzed with a log rank (Mantel Cox) test. Values were considered statistically different if *P* was <0.05. Data are representative of two independent experiments. Download Figure S2, PDF file, 0.1 MB

Figure S3  11H10_N297Q_ activity *in vitro* and *in vivo.* (A) OPK activity *in vitro*. *S. aureus* Newman was opsonized with serial dilutions of 11H10, 11H10_N297Q_, or c-IgG. Differentiated human monocytic cells (HL-60) were added at a 10:1 (HL60-to-bacterium) ratio, with human sera at a 1:100 dilution. The graph represents the means of results from three independent experiments ± standard deviations. (B) Inhibition of bacterial agglutination in human plasma. Serial dilutions of 11H10, 11H10N297Q, or c-IgG were mixed with human plasma, and bacterial agglutination was measured visually. Data are representative of three independent experiments. (C and D) MAb efficacy in i.v. lethal bacteremia. BALB/c mice (*n =* 10) were passively immunized i.p. with 11H10, 11H10_N297Q_, or c-IgG at 15 mg/kg and infected 24 h later with either *S. aureus* 3049043 (3e7 CFU) (C) or NRS382 (7e7 CFU) (D). Survival was monitored for 14 days. Statistical differences between survival for 11H10- and 11H10_N297Q_-immunized animals and that for c-IgG-immunized animals or between survival for 11H10-immunized animals and that for 11H10_N297Q_-immunized animals was analyzed with a log rank test. Statically significant differences are indicated with a number sign. Data are representative of two independent experiments. Download Figure S3, PDF file, 0.1 MB

Figure S4  The anti-AT–anti-ClfA combination provided broad strain coverage. BALB/c mice (*n =* 10) were immunized i.p. with MEDI4893* (15 mg/kg), 11H10 (15 mg/kg), MEDI4893* plus 11H10 (7.5 mg/kg each), or c-IgG (15 mg/kg). Twenty-four hours later, animals were infected i.v. in the tail vein with the LD_90_ (as indicated on each graph) of one of nine different *S. aureus* isolates from diverse clonal complexes (CC), and survival was monitored for 2 weeks. Statistical analysis was assessed with a log rank (Mantel Cox) test, and values were considered statistically significantly different from values for c-IgG-immunized animals if *P* was <0.05. Data are representative of at least three independent experiments. Download Figure S4, PDF file, 0.1 MB

Table S1 *In vitro* and *ex vivo* binding of anti-ClfA MAb 11H10 to 24 *S. aureus* clinical isolates. Table S1, PDF file, 0.1 MB

Table S2 Primers used for in-frame gene deletion of *clfA* using the pKOR1 system. Table S2, PDF file, 0.2 MB
