# Supplementary material for: Targeting Alpha Toxin and ClfA with a Multimechanistic Monoclonal-Antibody-Based Approach for Prophylaxis of Serious Staphylococcus aureus Disease
Source: mBio. 2016 Jun 28;7(3):e00528-16. doi: 10.1128/mBio.00528-16 (PMC4937210; doi:10.1128/mBio.00528-16)
Supplement: Figure S1 — 11H10 mediates OPK against multiple clinical isolates. Six different S. aureus clinical isolates were grown overnight in TSB medium. Bacteria were opsonized with serial dilutions of 11H10 or c-IgG as a negative control. Differentiated human monocytic cells (HL-60) were added at a 10:1 (HL60-to-bacterium) ratio with human sera at a dilution of 1:100 and with OPK activity measured. The graphs represent OPK killing as means of results from three separate experiments ± standard deviations. Download [file mbo003162872sf1.pdf]

FIG. S1

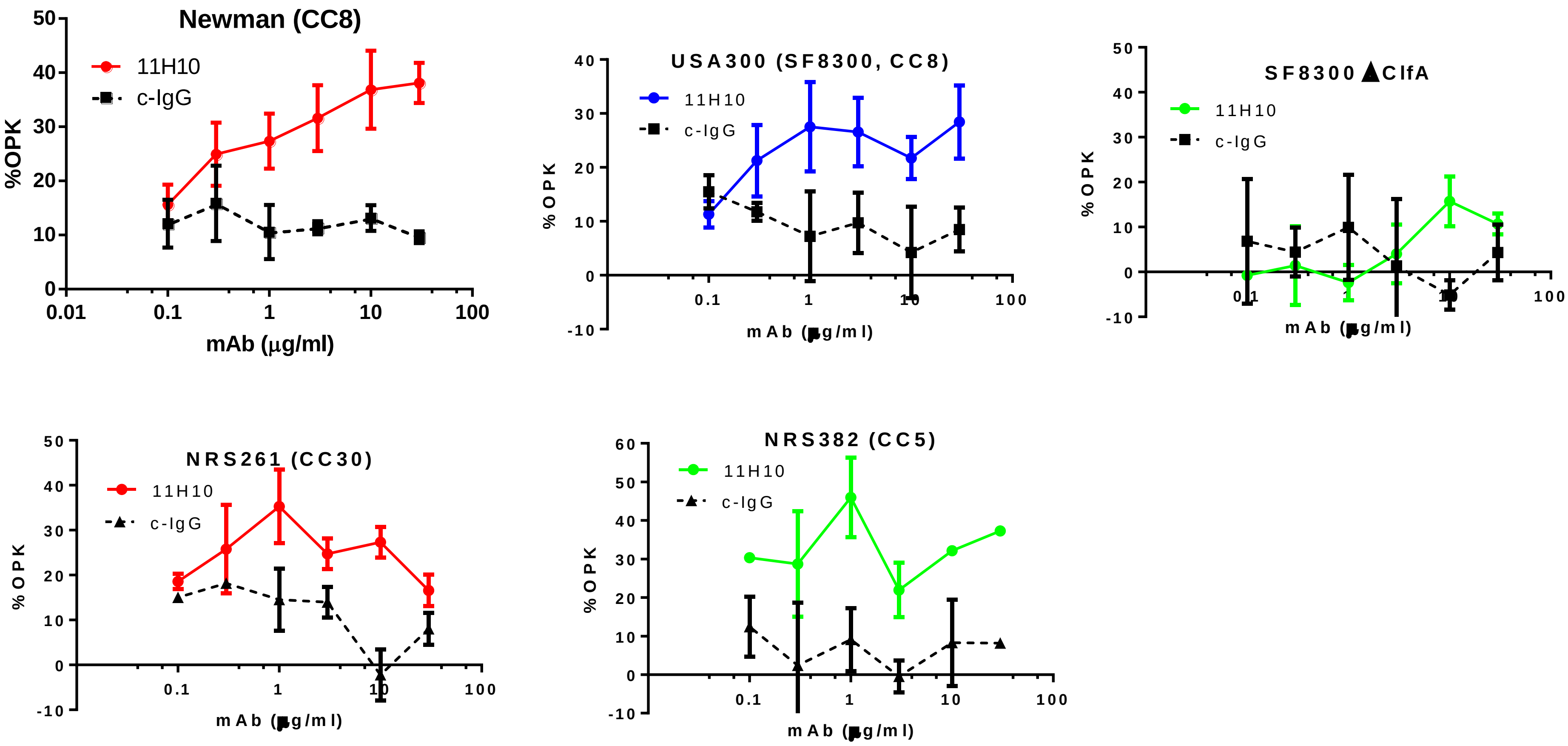

FIG S1 11H10 mediates OPK against multiple clinical isolates. Six different *S. aureus* clinical isolates were grown overnight in TSB medium. Bacteria were opsonized with serial dilutions of 11H10 or c-IgG as a negative control. Differentiated human monocytic cells (HL-60) were added at a 10:1 (HL60:bacteria) ratio with human sera at a dilution 1:100 and OPK activity measured. The graphs represent OPK killing as a mean of three separate experiments ± standard deviations.
