# Supplementary material for: Targeting Alpha Toxin and ClfA with a Multimechanistic Monoclonal-Antibody-Based Approach for Prophylaxis of Serious Staphylococcus aureus Disease
Source: mBio. 2016 Jun 28;7(3):e00528-16. doi: 10.1128/mBio.00528-16 (PMC4937210; doi:10.1128/mBio.00528-16)
Supplement: Figure S2 — Fc function is not required for MEDI4893* protection in lethal bacteremia. BALB/c mice (n = 10) were passively immunized i.p. with MEDI4893*, MEDI4893*N297Q, or c-IgG at 15 mg/kg and infected 24 h later with SF8300 (5e7 CFU). Survival was monitored for 14 days. Statistical differences between the survival of MEDI4893*- and MEDI4893*N297Q-immunized animals and that of c-IgG-immunized animals was analyzed with a log rank (Mantel Cox) test. Values were considered statistically different if P was <0.05. Data are representative of two independent experiments. Download [file mbo003162872sf2.pdf]

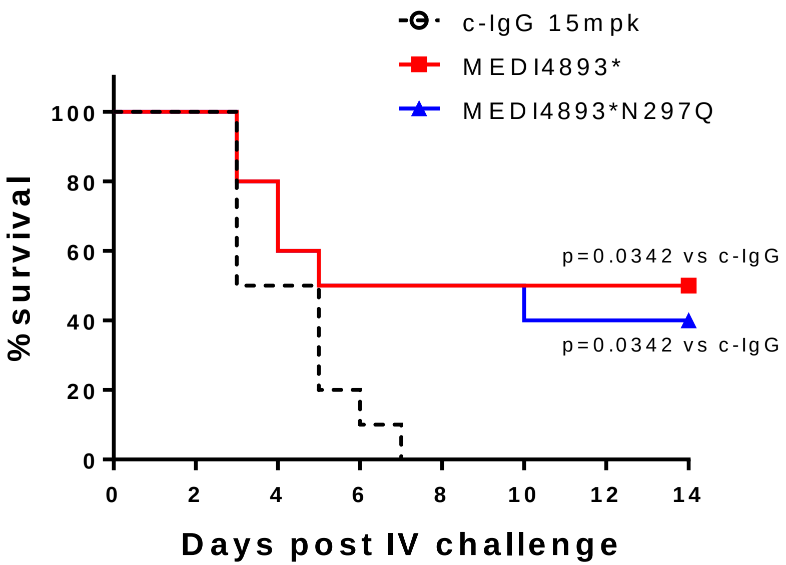

FIG S2 Fc function is not required for MEDI4893* protection in lethal bacteremia. Balb/c mice (n=10) were passively immunized IP with MEDI4893* (■), MEDI4893*$_{N297Q}$ (▲) or c-IgG (○) at 15mpk**,** and infected 24h later with SF8300 (5e7 CFU). Survival was monitored for 14 days. Statistical difference for survival between MEDI4893*, and MEDI4893*$_{N297Q}$ versus c-IgG was analyzed with a Log Rank (Mantel Cox) test. Values were considered statistically different if $p<0.05$. Data are representative of two independent experiments.
