# Supplementary material for: Targeting Alpha Toxin and ClfA with a Multimechanistic Monoclonal-Antibody-Based Approach for Prophylaxis of Serious Staphylococcus aureus Disease
Source: mBio. 2016 Jun 28;7(3):e00528-16. doi: 10.1128/mBio.00528-16 (PMC4937210; doi:10.1128/mBio.00528-16)
Supplement: Figure S3 — 11H10N297Q activity in vitro and in vivo. (A) OPK activity in vitro. S. aureus Newman was opsonized with serial dilutions of 11H10, 11H10N297Q, or c-IgG. Differentiated human monocytic cells (HL-60) were added at a 10:1 (HL60-to-bacterium) ratio, with human sera at a 1:100 dilution. The graph represents the means of results from three independent experiments ± standard deviations. (B) Inhibition of bacterial agglutination in human plasma. Serial dilutions of 11H10, 11H10N297Q, or c-IgG were mixed with human plasma, and bacterial agglutination was measured visually. Data are representative of three independent experiments. (C and D) MAb efficacy in i.v. lethal bacteremia. BALB/c mice (n = 10) were passively immunized i.p. with 11H10, 11H10N297Q, or c-IgG at 15 mg/kg and infected 24 h later with either S. aureus 3049043 (3e7 CFU) (C) or NRS382 (7e7 CFU) (D). Survival was monitored for 14 days. Statistical differences between survival for 11H10- and 11H10N297Q-immunized animals and that for c-IgG-immunized animals or between survival for 11H10-immunized animals and that for 11H10N297Q-immunized animals was analyzed with a log rank test. Statically significant differences are indicated with a number sign. Data are representative of two independent experiments. Download [file mbo003162872sf3.pdf]

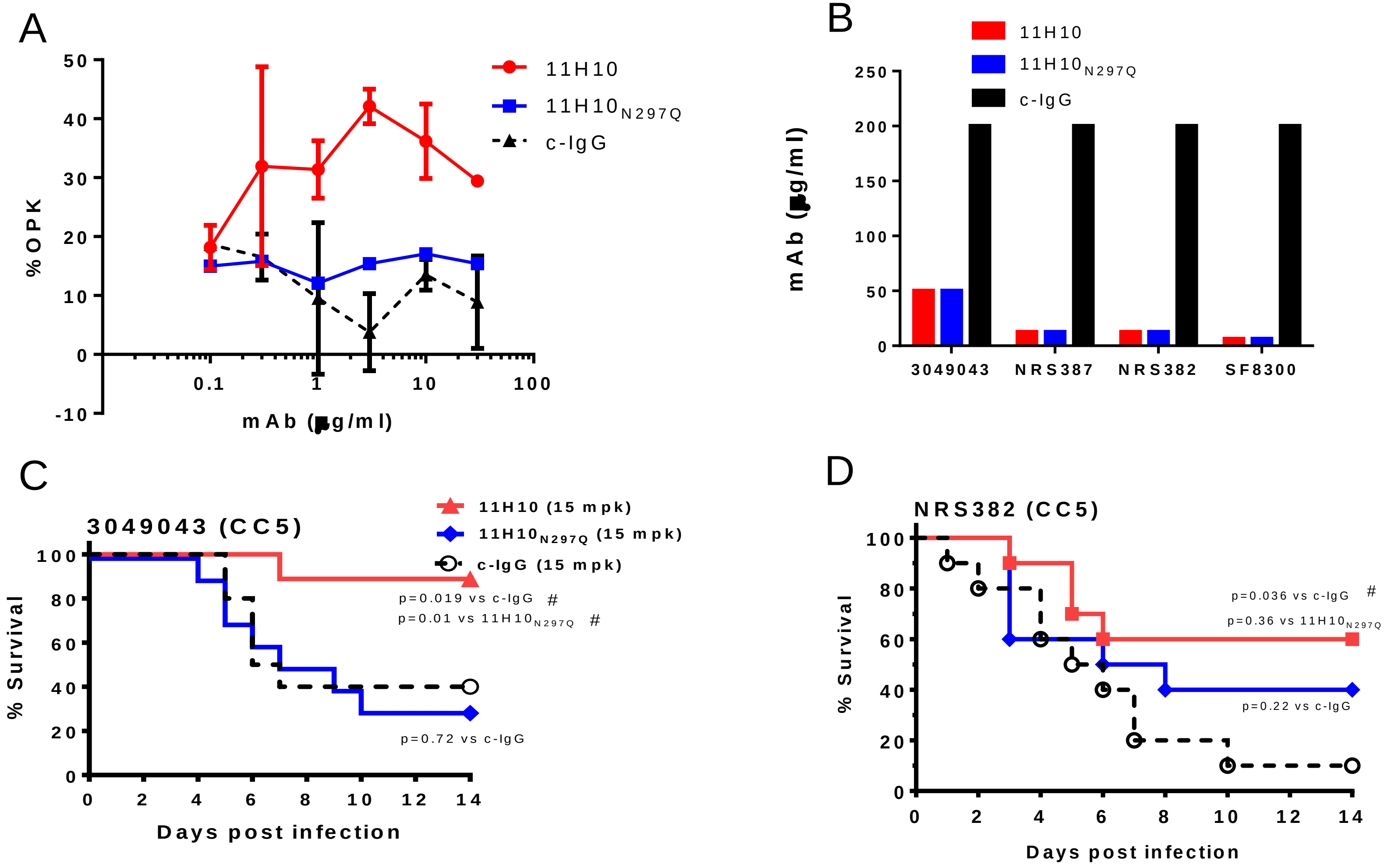

FIG S3 11H10$_{N297Q}$ activity in vitro and in vivo. (A) OPK activity in vitro. *S. aureus* Newman was opsonized with serial dilution of 11H10 (●), 11H10$_{N297Q}$ (■) or c-IgG (▲). Differentiated human monocytic cells (HL-60) were added at a 10:1 (HL60:bacteria) ratio, with human sera at a 1:100 dilution. The graph represents the mean of three independent experiments ± standard deviation. (B) Inhibition of bacteria agglutination in human plasma. Serial dilution of 11H10 (■), 11H10N297Q (■), or c-IgG (■) were mixed with human plasma and bacteria agglutination measured visually. Data are representative of three independent experiments (C and D) mAb efficacy in IV lethal bacteremia. Balb/c mice (n=10) were passively immunized IP with 11H10 (▲), 11H10$_{N297Q}$ (♦) or c-IgG (○) at 15mpk**,** and infected 24h later with either *S. aureus* 3049043 (3e7CFU) or NRS382 (7e7CFU). Survival was monitored for 14 days. Statistical difference for survival for 11H10, and 11H10$_{N297Q}$ versus c-IgG or 11H10 versus 11H10$_{N297Q}$ was analyzed with a Log Rank (Mantel Cox) test. Data were considered statistically different if $p<0.05$, and indicated with a (#) . Data are representative of two independent experiments.
