# Supplementary material for: Targeting Alpha Toxin and ClfA with a Multimechanistic Monoclonal-Antibody-Based Approach for Prophylaxis of Serious Staphylococcus aureus Disease
Source: mBio. 2016 Jun 28;7(3):e00528-16. doi: 10.1128/mBio.00528-16 (PMC4937210; doi:10.1128/mBio.00528-16)
Supplement: Figure S4 — The anti-AT–anti-ClfA combination provided broad strain coverage. BALB/c mice (n = 10) were immunized i.p. with MEDI4893* (15 mg/kg), 11H10 (15 mg/kg), MEDI4893* plus 11H10 (7.5 mg/kg each), or c-IgG (15 mg/kg). Twenty-four hours later, animals were infected i.v. in the tail vein with the LD90 (as indicated on each graph) of one of nine different S. aureus isolates from diverse clonal complexes (CC), and survival was monitored for 2 weeks. Statistical analysis was assessed with a log rank (Mantel Cox) test, and values were considered statistically significantly different from values for c-IgG-immunized animals if P was <0.05. Data are representative of at least three independent experiments. Download [file mbo003162872sf4.pdf]

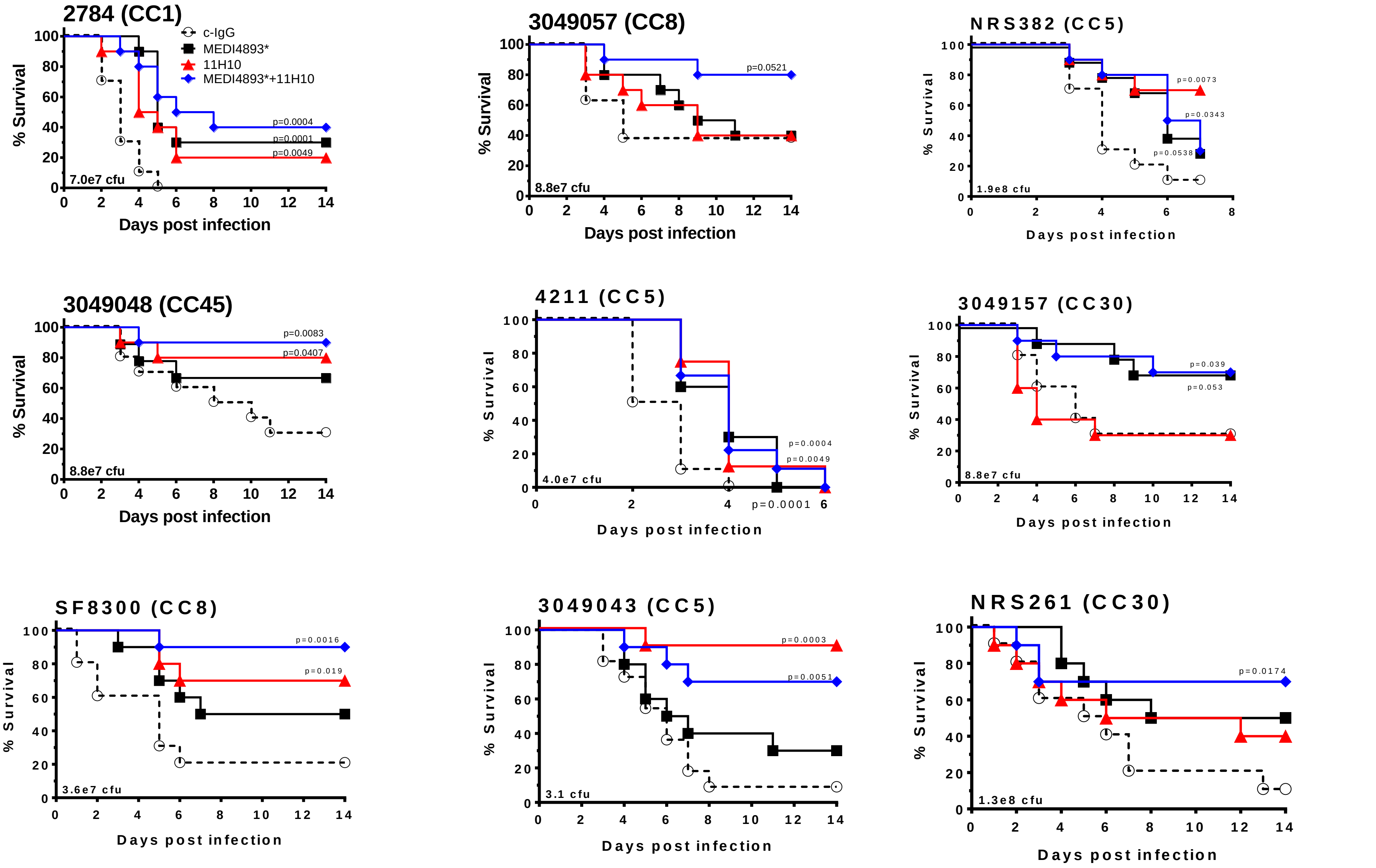

FIG S4 Anti-AT and anti-ClfA combination provided broad strain coverage. Balb/c mice (n=10) were immunized IP with 15mpk of MEDI4893* (■), or 11H10 (▲), MEDI4893*+11H10 (7.5mpk each) (♦) or c-IgG (15mpk) (○). Twenty-four hours later, animals were infected IV in the tail vein with LD_{90} (as indicated on each graph) of one of nine different *S. aureus* isolates from diverse clonal complexes (CC) and survival monitored for 2 weeks. Statistical analysis was assessed with a Log Rank (Mantel-Cox) test, and considered statistically different versus c-IgG if $p<0.05$. Data are representative of at least three independent experiments.
