# Supplementary material for: Targeting Alpha Toxin and ClfA with a Multimechanistic Monoclonal-Antibody-Based Approach for Prophylaxis of Serious Staphylococcus aureus Disease
Source: mBio. 2016 Jun 28;7(3):e00528-16. doi: 10.1128/mBio.00528-16 (PMC4937210; doi:10.1128/mBio.00528-16)
Supplement: Table S1 — In vitro and ex vivo binding of anti-ClfA MAb 11H10 to 24 S. aureus clinical isolates. [file mbo003162872st1.pdf]

**Table S1:** In vitro and ex vivo binding of anti-ClfA mAb11H10 to 24 *S. aureus* clinical isolates.

Clinical isolates from different clonal complexes (CC) or unknown CC (UNK) were injected IP into CD1 mice (5e8CFU/mouse) and blood collected 1 or 4h later. 11H10 binding was measured by cytofluorimetry and compared with c-IgG staining. Binding was also measured by cytofluorimetry on bacteria grown overnight in TSB. (+) indicates >50% of bacteria exhibited at least a one log shift in mean fluorescence as compared to c-IgG. Data represent binding from two independent experiments.

| | NRS123 | ARC634 | ARC635 | ARC797 | ARC2379 | NRS382 | NRS384 | ARC2464 | ARC517 | SF8300 | Newman |
|---|---|---|---|---|---|---|---|---|---|---|---|
| CC | 1 | 5 | 5 | 5 | 5 | 5 | 8 | 8 | 8 | 8 | 8 |
| In vitro | - | - | - | + | - | - | - | - | + | + | + |
| 1h post | + | + | + | - | + | + | + | + | + | + | + |
| 4h post | - | + | - | + | - | - | - | - | - | + | + |

| | BAA1556 | NRS249 | NRS234 | ARC633 | UAMS-1 | ARC2081 | NRS22 | ARC516 | ARC2558 | ARC1056 | NRS655 | ARC2558 |
|---|---|---|---|---|---|---|---|---|---|---|---|---|
| CC | 8 | 247 | 15 | 15 | 30 | 30 | 45 | UNK | UNK | UNK | UNK | UNK |
| In vitro | - | - | - | + | + | + | + | + | - | + | + | - |
| 1h post | - | - | - | + | + | + | + | + | + | + | + | + |
| 4h post | + | + | + | - | + | + | + | + | + | + | + | + |
