# Supplementary material for: Targeting Alpha Toxin and ClfA with a Multimechanistic Monoclonal-Antibody-Based Approach for Prophylaxis of Serious Staphylococcus aureus Disease
Source: mBio. 2016 Jun 28;7(3):e00528-16. doi: 10.1128/mBio.00528-16 (PMC4937210; doi:10.1128/mBio.00528-16)
Supplement: Table S2 — Primers used for in-frame gene deletion of clfA using the pKOR1 system. [file mbo003162872st2.pdf]

Table S2: Primers used for in-frame gene deletion of clfA using the pKOR1 system.

Primer ID	5' → 3' nucleotide sequence
clfA-X1	ggaggggcaaagatgc
clfA-X2	CGTATTTGCTTCATCTTCAGAACCACCTACAAGCACTGAAGCCAC
clfA-X3	GTGGCTTCAGTGCTTGTAGGTGGTTCTGAAGATGAAGCAAATACG
clfA-X4	GCTTCAGTGTCAGATTTTAATTGAGC
clfA-X5	GGGG ACAAGTTTGTACAAAAAAGCAGGCT ctttgctcaagtggaccag
clfA-X6	GGGG ACCACTTTGTACAAGAAAGCTGGGT GTCGCACTTTAATTGCTCCTC
clfA-S1	gtttgcaatcaaatcgtacgttgtc
clfA-S2	GAAACCACTGCACTCGCAC
